# Investigating the multivariate nature of NHL player performance with structural equation modeling

**DOI:** 10.1371/journal.pone.0184346

**Published:** 2017-09-08

**Authors:** Sean N. Riley

**Affiliations:** Institute of Cognitive Science, Carleton University, Ottawa, Ontario, Canada; University of Münster, GERMANY

## Abstract

Hockey is a complex and multifaceted game, yet many of the statistical tools used to evaluate performance are univariate. To garner a better understanding of hockey’s multifaceted nature, two structural equation models (SEMs) assessing the interrelations between offense, defense, and possession were built from three seasons of NHL data. Overall, it was found that the concepts of offense, defense, and possession are best understood via a small constellation of measured variables, and that offense mediates the relationship between possession and defense such that higher levels of offense leads to poorer defensive performance. These findings are discussed within the context of ranking player performance.

## Introduction

### What is ice-hockey?

Ice-hockey (referred to as hockey for the remaining text) is a complex, fast-paced, team versus team sport whereby each team tries to shoot a small puck into a net more times than their opponent (each instance of which is referred to as a “goal”). Teams are allowed to have six players on the ice at any given time (typically three forwards, two defensemen, and one goaltender), with the game being played in three, 20 minute, stop-time periods. Stoppages in play occur when (i) a rule is broken, (ii) the goaltender covers the puck, (iii) the puck goes out the defined playing area, or (iv) a goal is scored. Like other professional sports, there are different “levels” at which the game is played, the highest being the National Hockey League (NHL), which involves 30 teams spread across Canada and the United States.

### Sport analytics

Since Bill James’ seminal work on sabermetrics (a set of statistical tools to assess team and player performance in baseball), there has been a growing interest in the empirical analysis of sport [1–3]. These types of analyses appear to have value in relatively slow-paced sports such as baseball and golf [4, 5], as well as relatively fast-paced sports such as basketball, football, and American football [6–8]. Yet despite their effectiveness and wide-spread adoption, hockey has been relatively slow to develop specialized data analysis tools.

Recent efforts in this regard have brought about a wide range of descriptive statistics, with the entire corpus being referred to as “advanced statistics” by the hockey community. Fortunately, the majority of these advanced statistics fit within a hierarchical structure such that upward movement produces an increase in specificity, with this specificity being geared towards capturing the complex, interactive effects prevalent within the game (see also [9]).

### Hockey performance metrics

#### Level 1: Raw performance metrics

At the most fundamental level sits raw performance metrics such as goals, assists, shots, and-so-forth. Although the number and variety of advanced statistics at this level is vast, this paper focuses on a small number of metrics: corsi, points, goals for, goals against, assists, and faceoff location.

**Defintition 1**. Corsi is the total number of shots that (i) were on net, (ii) missed the net, or (iii) were blocked on route to the net.

Corsi values can be broken down into a number of different metrics, such as corsi for (corsi events against the opposing team), corsi against (corsi events against the player’s team), corsi for percentage (corsi for divided by the sum of corsi for and corsi against). Further, each of these metrics can be broken down according to a variety of different grouping values (e.g, per 60 minutes of icetime). Finally, corsi is generally measured at the linemate level as individual corsi metrics are captured by other measures (e.g., shots, shot attempts etc.) Thus, anytime a corsi event occurs, that event is recorded for every player on the ice. Unfortunately, there exists no standardized symboling system for corsi (or any other advanced statistic) within the academic literature, so I will adopt the following:

Time on ice = TOI
Corsi = $C_{metric}^{grouping}$
Corsi for = *C*_*f*_
Corsi against = *C*_*a*_
Corsi for percent = *C*_*p*_ = *C*_*f*_/(*C*_*f*_ + *C*_*a*_)
Corsi for per 60 = $C_{f}^{60}$ = 60(*C*_*f*_/*TOI*)
Corsi against per 60 = $C_{a}^{60}$ = 60(*C*_*a*_/*TOI*)

**Defintition 2**. Goals are the number of times the puck is shot past the goalie and into the net.

As is the case with corsi, goals can be broken down by metric and grouping:

Goals = $G_{metric}^{grouping}$
Individual goals for = *G*_*i*_
Goals for while on the ice (WOI, scored by player or linemate) = *G*_*f*_
Goals against WOI = *G*_*a*_
Goals for WOI per 60 = $G_{f}^{60}$
Goals against WOI per 60 = $G_{a}^{60}$
Individual goals for per 60 = $G_{i}^{60}$

**Defintition 3**. An assist on a goal is awarded to a maximum of two players on the scoring team, not including the goal scorer, that touched the puck in a way that helped facilitate the goal, be it by shooting, passing, or deflecting the puck.

Assists = $A_{metric}^{grouping}$
Individual assists = *A*_*i*_
Individual assists per 60 = $A_{i}^{60}$

**Defintition 4**. Points are the number of goals plus the number of assists.

Points = $P_{metric}^{grouping}$
Individual points = *P*_*i*_
Individual points per 60 = $P_{i}^{60}$

#### Level 2: Relative to team

The focus at this level is on taking raw metrics and situating them within the context of the entire team [9]. For example, if one wanted to see a player’s *C*_*p*_ relative to the rest of their team, all one would have to do is take that player’s *C*_*p*_ and subtract the *C*_*p*_ of the team when the player is not on the ice.

**Defintition 5**. Off-ice metrics for a player are the metrics posted by the team when the player is not on the ice during games the player participates in.

**Defintition 6**. Relative to team metrics are on-ice metrics minus off-ice metrics.

I have elected to signal these metrics by placing *τ* before the raw metric:

Corsi for percent relative to team = *τC*_*p*_
Corsi for per 60 relative to team = $\tau C_{f}^{60}$
Corsi against per 60 relative to team = $\tau C_{a}^{60}$
Goals for per 60 relative to team = $\tau G_{f}^{60}$
Goals against per 60 relative to team = $\tau G_{a}^{60}$

It should be noted that not every raw metric has an associated *τ*-metric, thus *τ*-metrics typically make use of raw metrics involving percentages or standardized groupings (e.g, per *x* minutes of ice-time).

Overall, the goal of *τ*-metrics is to get an idea of whether a player helps or hinders their team’s overall performance. If a player has a positive $\tau G_{f}^{60}$, then that tells us something important about that player’s impact on their team, namely that the team scores a higher rate of goals when the player is on the ice than when the player is off the ice.

#### Level 3: Relative to linemates

As beneficial as *τ*-metrics are, it is also helpful to know how a player performs relative to their linemates. For example, if we wanted to see how a player’s $C_{f}^{60}$ differs from their linemates, we would first find every linemate the player has had over the course of the season, then calculate each linemate’s $C_{f}^{60}$ for the time they are *not* on the ice with the player. Next, we weigh that $C_{f}^{60}$ by the amount of time they *did* spend on the ice with the player. Once we have weighted values for each linemate, we simply take the average and subtract it from the player’s $C_{f}^{60}$ [9].

**Defintition 7**. Relative to linemate metrics are a player’s raw metric, minus the weighted average of their linemates’ raw metric while playing on a different line.

This approach is needed to strip away as much of the interaction between players as possible. That is, individual player performance is highly dependent on linemate performance; thus, the thinking goes, because the player’s $C_{f}^{60}$ contains within it their individual performance *and* linemate interaction, if we take out linemate performance while playing on a different line, we are, in effect, taking out the contribution of linemates to the player’s performance. Otherwise stated: if, on average, a player’s linemates perform better when they are on a different line, then that player is, on average, worse than their linemates and drags down their linemates performance. Obviously this is not an idea formulation as the interactive effects of linemates are more than the sum of their individual parts, but it does provide us with a rough estimate.

I have elected to indicate relative to linemate metrics by preceding the raw metric with *δ*.

Corsi for percent relative to linemates = *δC*_*p*_
Corsi for per 60 relative to linemates = $\delta C_{f}^{60}$
Corsi against per 60 relative to linemates = $\delta C_{a}^{60}$
Goals for per 60 relative to linemates = $\delta G_{f}^{60}$
Goals against per 60 relative to linemates = $\delta G_{a}^{60}$

### Prior research on hockey analytics

As previously noted, hockey has lagged behind other sports with respect to data analysis; however, some interesting results have still been produced.

For example, Macdonald [10] used a variety of raw metrics (goals, shots, hits, hits against, and faceoffs) to build a ridge regression model predicting the number of goals a player would score in the future. All told, their model produced a correlation between actual and predicted goals of 0.69, and performed better than any of the raw metrics did individually. Perhaps more interesting was that corsi produced the highest correlation (0.51) of any of the raw metrics, which suggests corsi (and by virtue puck possession as you need to have possession of the puck if you want to shoot it), is a key variable of interest.

In a similar vein, Thomas and colleagues [11] modeled goal scoring as a semi-Markov process, and in the course of their investigation found that player performance is greatly influenced by the interactions between a player and their linemates. For example, despite Sidney Crosby and Evgeni Malkin being two of the best individual players in the world, when they played together their performance did not improve, and actually led to more goals against [11]. Conversely, when Brad Boyes and Jay McClement played together, they both performed at a level beyond their individual abilities [11].

These findings are paralleled by the work of Gramacy and colleagues [12], who built a regularized logistic regression model of players’ individual contributions to their team’s goal scoring. Overall, the regression model served as a way to expand on the traditional plus-minus statistic (which is calculated as *G*_*f*_ − *G*_*a*_) by controlling for the contributions of teammates, and found that a relatively narrow band of players had a significant effect on goal scoring, be it positive or negative [12].

The idea of quantifying individual performance was taken a step further by Schuckers and Curro’s Total Hockey Rating (THoR), which is based on (i) every non-shooting on-ice event for a player, (ii) whether the player had home-ice advantage, (iii) what zone the play started in, and (iv) everyone else that was on the ice with the player [13]. The model was fit using ridge regression, with the THoR giving us an estimate of the number of wins created by a player over the course of an 82 game season [13]. Overall, it was found that forwards are, typically, responsible for more wins created than defensemen, with elite players producing over five wins per season [13].

Shifting away from individual performance, work by Roith and Magel [14] demonstrated that, given a full season of data, only the total number of goals against, the total number of goals for, and the total number of takeaways are needed to accurately predict (87%) whether a team would make or miss the playoffs. Moreover, the authors presented a logistic regression model predicting which team would win a given game, and found that only a handful of variables pertaining to shots, faceoffs, and save percentage were needed to accurately predict the winner (which further highlights the importance of corsi metrics in understanding NHL player performance) [14].

Additional efforts have been made to classify NHL players based on their style of play [15, 16], as well developing visualization techniques to assess the various spatial properties of the game [17, 18]. However, as beneficial as the aforementioned research is, it has largely relied on univariate regressions; that is, even though there are multiple independent variables, there is only one dependent variable. Although these univariate methods are valuable when the domain is limited to a single measure such as goals, core concepts such as offense and defense cannot be fully captured by a single measure. Moreover, univariate techniques do not allow for systems of regression equations; this is problematic as it does not allow a measure to simultaneously be a regressor and a regressand, which means the structural relationships between multiple measures cannot be assessed in a single model (e.g. the way possession, offense, and defense all effect one another) [19].

### Structural equation modeling

Structural equation modeling (SEM) is a relatively new, and increasingly popular, statistical technique designed to address the issues outlined above by combining factor analysis with tools such as regression and analysis of variance [19].

At its core, a SEM consists of two categories of variables (measured and latent) and a path diagram that specifies the relationships between these variables [19–21]. Here, the idea is that some constructs cannot be fully captured by a single measured variable. For example, the construct of offense in hockey cannot be fully captured by points alone (a player with 20 goals and 80 assists is very different than a player with 80 goals and 20 assists), but rather exists as some combination of multiple measured variables (e.g. points, goals, assists, and-so-on). Thus, measured variables in SEM are variables that one has observed and directly collected data on, with latent variables being unobserved variables that are inferred from measured variables (e.g. offense as inferred from goals, assists, points, and-so-on) [19, 22]. The relationship between measured and latent variables is determined via a confirmatory factor analysis (CFA), with each latent variable being a linear combination of its measured variables [22]. These relationships can then be used to compute factor scores for latent variables, which gives us a measure of how well a person scores on each latent variable [23]. The relationship between all of the measured variables and all of the latent variables is called the measurement model; conversely, the path diagram is referred to as the structural model, and specifies the relationships between latent variables as calculated by a system of regressions, ANOVAs, or other similar techniques [19, 21, 24].

One significant benefit of using SEM to analyze hockey data is SEM’s ability to deal with multicollinearity. As discussed by MacDonald [10] and Gramancy et al. [12], NHL performance metrics are often highly correlated, which introduces problems in univariate regression. The problem of multicollinearity can be addressed in univatiate models by using techniques such as ridge regression; however, in SEM, these measured variables are represented as a single factor (a latent variable) that presumes measured variables are highly correlated (if they were not, then they would not represent the same latent variable), thus the problem of multicollinearity is averted altogether [25].

Similarly, SEM’s use of latent variables and its ability to easily specify multivariate models has made it a popular tool in fields that infer characteristics based on multiple measured variables [19]. Given that core concepts in hockey such as offense, defense, and possession are best understood in terms of multiple measured variables, SEM affords us the unique ability to assess how all of these measured variables impact one another, something that is currently lacking in the literature. That said, it is important to note that a SEM is not a causal model, and is only meant to determine (i) the factor structure of latent variables, and (ii) if latent variables have direct and/or indirect effects on each other [19, 25]. Of course, the problem of causality also arises in univariate models, and is an unfortunate byproduct of this field of research.

Overall, the goal of SEM is to specify a model whose estimated means and covariances (referred to as parameter estimates) fit the observed data. If a model produces parameter estimates that closely match the data, that model is said to be accepted; if the parameter estimates do not match the data, then the model is said to be rejected.

### Aims of current research

The univariate nature of prior research runs counter to the multivariate nature of hockey; offense cannot be fully captured by a single measure such as goals or points, nor can possession be fully captured by corsi for percentage, nor defense by goals against. Moreover, the concepts of offense, defense, and possession are best described by a constellation of measured variables, thus it is beneficial if assessments of performance include enough measured variables to sufficiently capture the concepts in question. With this in mind, the aim of this research is to identify a system of regressions and a constellation of measured variables that fit both the data and prior research. Extending the work of Macdonald [10] and Thomas et al. [11], I propose that only a small number of measured variables are needed to sufficiently capture the multivariate concepts of offense, defense, and possession, and that a system of regressions whereby offense acts as a mediator between possession and defense will generate parameter estimates that fit the data.

## Materials and methods

### Data used

To fit the model, I make use of three seasons worth of NHL data (2012/2013 to 2014/2015) retrieved from a well-known public repository compiled from official game reports supplied by the NHL (note: this repository, www.puckalytics.com, has since shut down as the website owner has been hired by an NHL team. Additional repositories can be found here: [26]). I limited data to even-strength situations (when both teams had five skaters and one goaltender on the ice), and to players who had combined for at least 200 minutes of icetime over the three seasons of interest. These limitations were selected because (i) power-play and penalty-kill situations are relatively rare and require major changes to on-ice strategy, and (ii) a limited sample of icetime is unlikely to produce reliable performance data, and using a 200 minute threshold removed players who, for whatever reason (e.g. injury), only played in a small number of games. Overall, the dataset consisted of 678 players who had between 200.80 and 1735.97 minutes of icetime (*M* = 843.98, *SD* = 334.83).

### Model

In an attempt to build a more complete picture of how popular advanced statistics related to each other, I built two SEMs with the same structural model, but differing measurement models.

The measurement model of the first SEM can be seen in Table 1. This is then compared against a second measurement model (Table 2) that includes additional measured variables, specifically individual goals for per 60 ($G_{i}^{60}$), individual assists per 60 ($A_{i}^{60}$), offensive zone faceoff percentage (the percentage of faceoffs that occur in the offensive zone; *OZFO*_*p*_), and defensive zone faceoff percentage (the percentage of faceoffs that occur in the defensive zone; *DZFO*_*p*_). $G_{i}^{60}$ and $A_{i}^{60}$ were selected because they provided detailed information above-and-beyond $P_{i}^{60}$. Similarly, *OZFO*_*p*_ and *DZFO*_*p*_ were selected on the grounds that faceoff metrics have been linked to both team and player performance [13, 14]. *OZFO*_*p*_ was placed under *possession* as it was found to produce a better model fit than when under *offense*, with *DZFO*_*p*_ being placed under *defense* as it produced a better model fit than when under *possession*. Although seemingly contradictory (would faceoff location not be an indicator of *possession*?), this phenomenon can possibly be explained by icings, and how the team that ices the puck is not allowed to substitute players. This can lead to tired skaters who may be more likely to make a defensive mistake that leads to a goal against, thus placing *DZFO*_*p*_ under *defense*, as opposed to *possession*.

**Table 1:** Measurement model 1.

Latent Variable	Measured Variables
*Possession*	*C*_*p*_, *τC*_*p*_, *δC*_*p*_
*Offense*	$P_{i}^{60}$, $G_{f}^{60}$, $\tau G_{f}^{60}$, $\delta G_{f}^{60}$
*Defense*	$G_{a}^{60}$, $\tau G_{a}^{60}$, $\delta G_{a}^{60}$

**Table 2:** Measurement model 2.

Latent Variable	Measured Variables
*Possession*	*C*_*p*_, *OZFO*_*p*_, *τC*_*p*_, *δC*_*p*_
*Offense*	$P_{i}^{60}$, $G_{i}^{60}$, $A_{i}^{60}$, $G_{f}^{60}$, $\tau G_{f}^{60}$, $\delta G_{f}^{60}$
*Defense*	$G_{a}^{60}$, *DZFO*_*p*_, $\tau G_{a}^{60}$, $\delta G_{a}^{60}$

**Fig 1:**
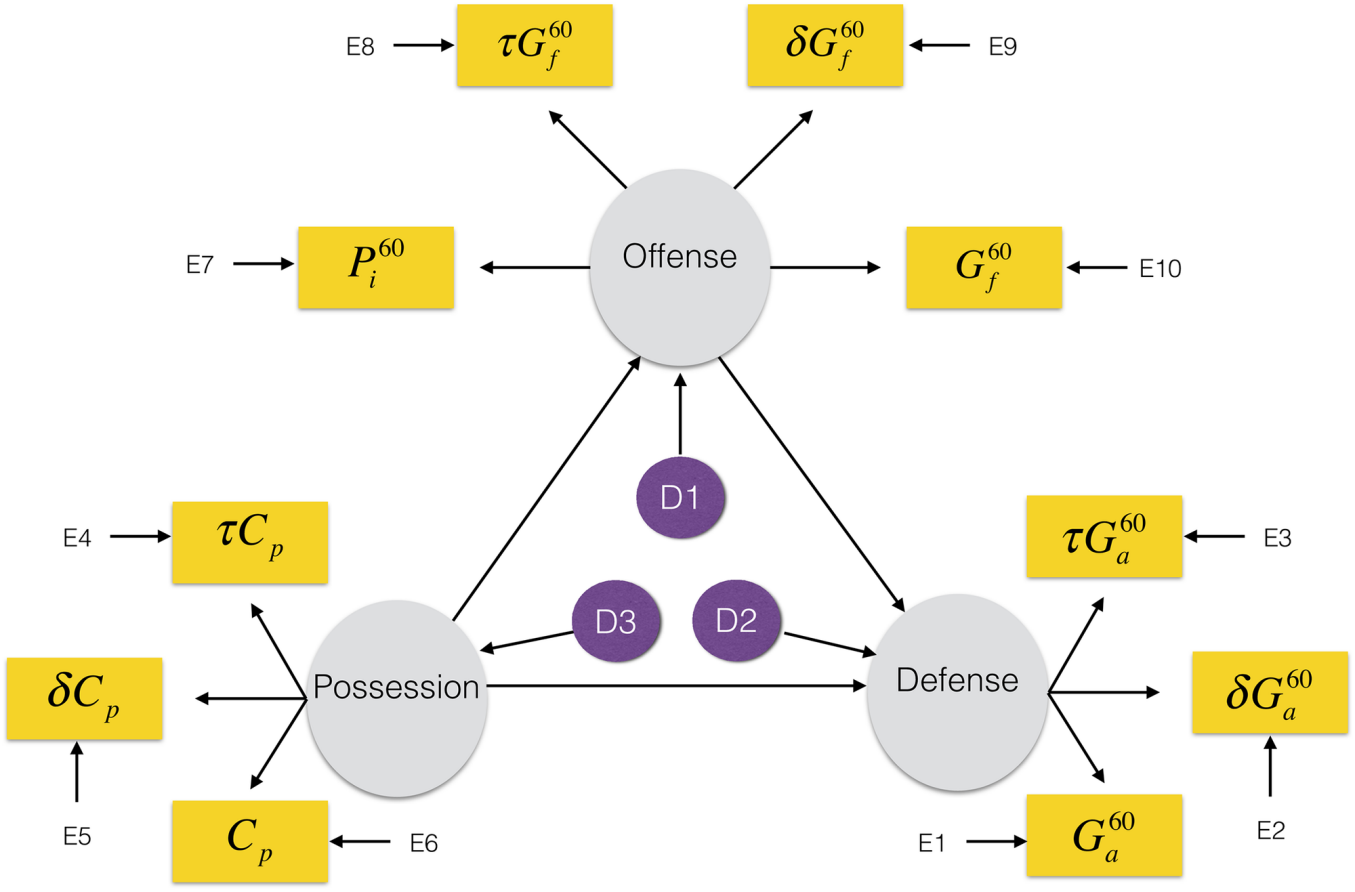
SEM diagram. The structural model with measurement model 1. Grey circles are latent variables, purple circles are disturbances, with yellow boxes being measured variables.

The structural model (Fig 1) has paths from *possession* to *offense*, *possession* to *defense*, and from *offense* to *defense*. Further, all latent variables have disturbances to account for any unspecified predictors. These disturbances are uncorrelated under the premise that *defense* disturbances can largely be attributed to goaltender skill, which has no impact on offense; and that *offense* disturbances can largely be attributed to individual skills such as shooting percentage (how often a shot leads to a goal), which have no bearing on defense. Moreover, *possession* disturbances can largely be attributed to metrics such as offensive zone faceoff win percentage and offensive zone entry metrics, which have no bearing on the skill metrics of *offense* and *defense*. Finally, disturbances are not removed under the second measurement model as the additional measured variables do not comprise an exhaustive list of all the measured variables that comprise each latent variable.

The theory behind each SEM is simple: (i) if a team/line/player spends more time in possession of the puck, then they are not only more likely to score more goals/points, but also have fewer goals scored against them; and (ii) players/lines with a high level of offensive output are more likely to have goals scored against them (possibly) due to missed defensive coverages brought about by an overemphasis on offense.

## Results

All analyses were conducted in R, and made use of the lavaan package for structural equation modeling (using a maximum likelihood estimator) [27].

### Descriptives

Descriptives statistics for measured variables can be seen in Table 3. Using a cut off of ±1 for skew and ±3 for kurtosis, all of our measured variables were normally distributed except for two kurtosis violations: $\tau G_{a}^{60}$ (3.85) and *DZFO*_*p*_ (5.14), which suggests a large number of players’ scores for $\tau G_{a}^{60}$ and *DZFO*_*p*_ clustered about the mean. Overall, the high level of univariate normality exhibited by the data means it is unlikely the models will produce biased parameter estimates that deviate from observed scores.

**Table 3:** Descriptives for measured variables.

	*M*	*SD*	Min	Max	Skew	Kurtosis
*C*_*p*_	49.65	3.79	37.34	61.25	-0.09	-0.03
*τC*_*p*_	-0.32	3.65	-15.10	9.18	-0.38	0.35
*δC*_*p*_	-0.04	3.29	-14.38	9.08	-0.25	0.45
*OZFO*_*p*_	31.73	5.53	4.81	52.10	-0.25	1.63
$P_{i}^{60}$	1.12	0.56	0.00	2.70	0.36	-0.60
$A_{i}^{60}$	0.69	0.34	0.00	1.98	0.51	0.19
$G_{i}^{60}$	0.43	0.32	0.00	1.39	0.56	-0.55
$G_{f}^{60}$	2.09	0.56	0.47	3.83	-0.08	-0.11
$\tau G_{f}^{60}$	-0.07	0.69	-2.41	2.05	-0.03	-0.13
$\delta G_{f}^{60}$	-0.03	0.59	-1.49	1.75	0.17	-0.16
$G_{a}^{60}$	2.15	0.46	0.88	5.21	0.57	2.67
$\tau G_{a}^{60}$	0.00	0.53	-1.76	3.86	0.51	3.85
$\delta G_{a}^{60}$	-0.03	0.50	-1.71	3.01	0.37	1.70
*DZFO*_*p*_	31.96	5.42	9.29	64.91	1.00	5.14

### Assumption testing

#### Multivariate normality

Mardia’s multivariate normality tests revealed that none of our latent variables were multivariate normal (Table 4). However, prior research on SEM suggests that violating multivariate normality does not undermine findings. For example, there is compelling evidence that maximum likelihood estimation is robust to normality violations, especially when sample sizes are large (e.g., *N* > 600), such as in this study (*N* = 678) [20, 21, 28, 29]. Moreover, as Winston and Gore [24] point out, normality should be evaluated at the univariate level as demonstrating multivariate normality requires examining an infinite number of linear combinations of variables [24]. Further work by Muthen and Kaplen [30] found that violations of multivariate normality had a negligible impact on parameter estimates and fit statistics, except in cases of extreme violations of both multivariate kurtosis and multivariate skew, in which case rates of model rejection actually increased. These findings are echoed by Hallow [31], who found that violations of univariate and/or multivariate normality produced unbiased parameter estimates, and by Curran and colleagues [32], who found that non-normality produced an overestimated chi-square test statistic, which makes model rejection more likely. However, as Henly [33] points out, samples smaller than *N* = 300 produce biased parameter estimates that lead to greater rates of model rejection, and that non-normal samples should be *N* > 600 to ensure unbiased parameter estimates.

**Table 4:** Mardia’s multivariate skew and kurtosis.

	Latent Variable	χ^2^ (Skew)	p-value	Z-value (Kurtosis)	p-value
Model 1					
	*Possession*	44.09 (0.39)	p <.01	9.99 (19.20)	p <.01
	*Offense*	152.52 (1.35)	p <.01	2.95 (25.57)	p <.01
	*Defense*	123.40 (1.09)	p <.01	17.48 (22.35)	p <.01
Model 2					
	*Possession*	85.96 (0.76)	p <.01	11.68 (30.21)	p <.01
	*Offense*	277.88 (2.46)	p <.01	52.41 (5.86)	p <.01
	*Defense*	269.59 (2.39)	p <.01	21.95 (35.68)	p <.01

**Fig 2:**
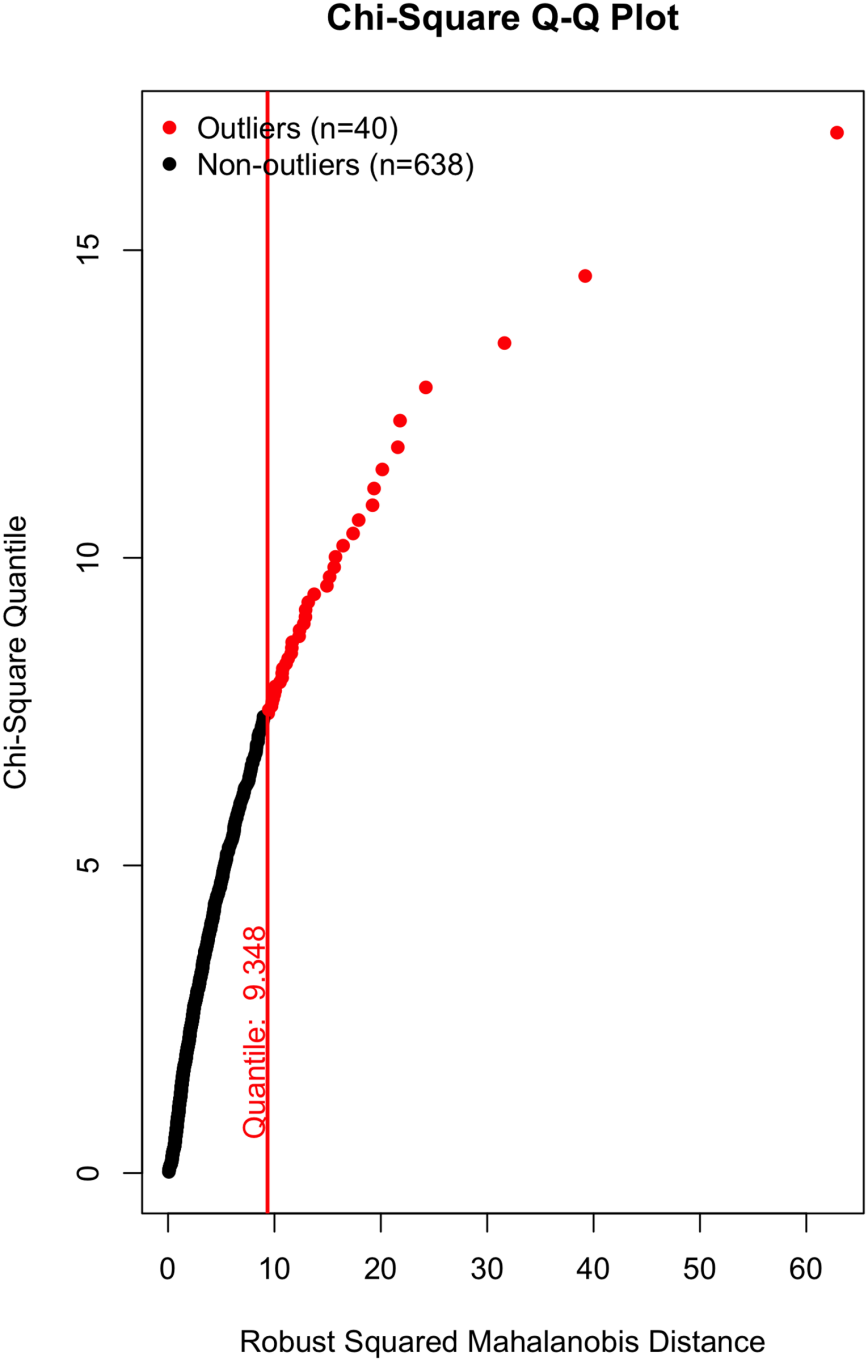
Multivariate outliers. Possession in Model 1.

**Fig 3:**
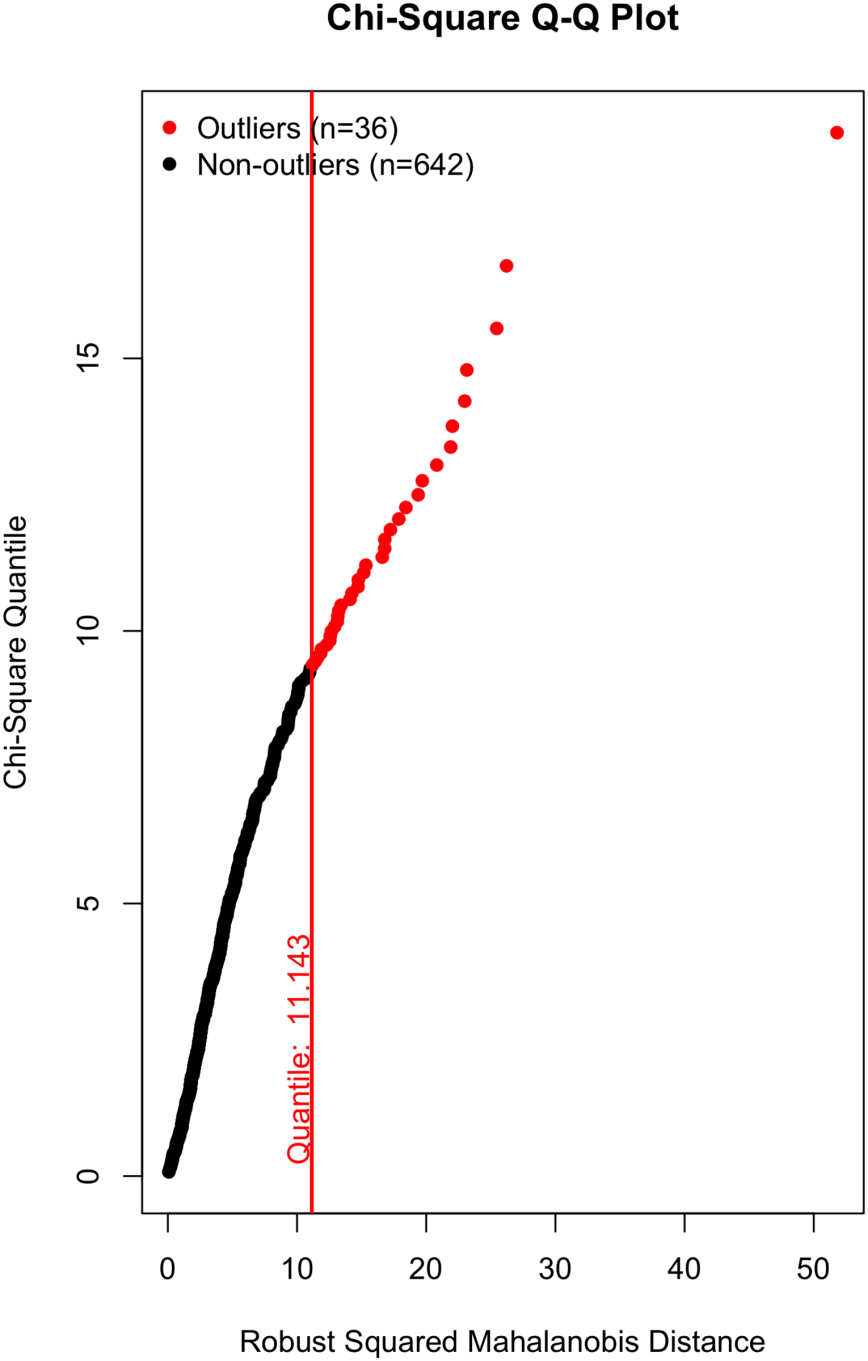
Multivariate outliers. Offense in Model 1.

**Fig 4:**
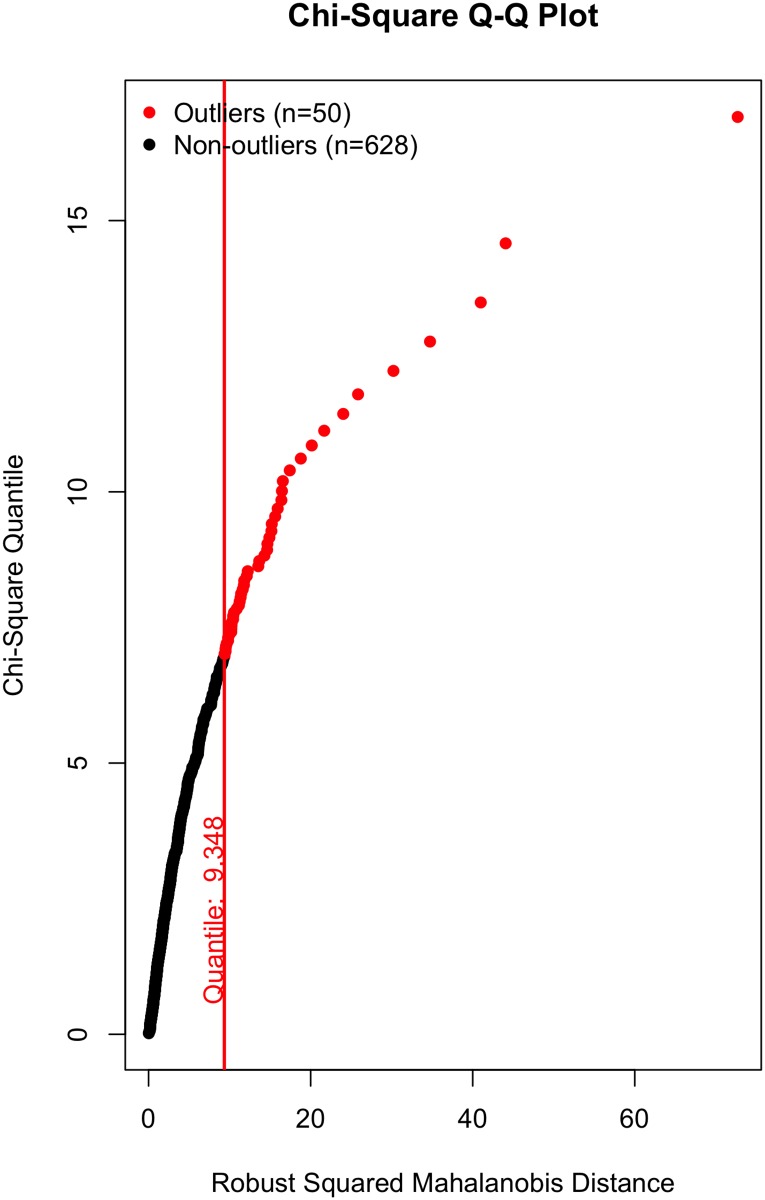
Multivariate outliers. Defense in Model 1.

**Fig 5:**
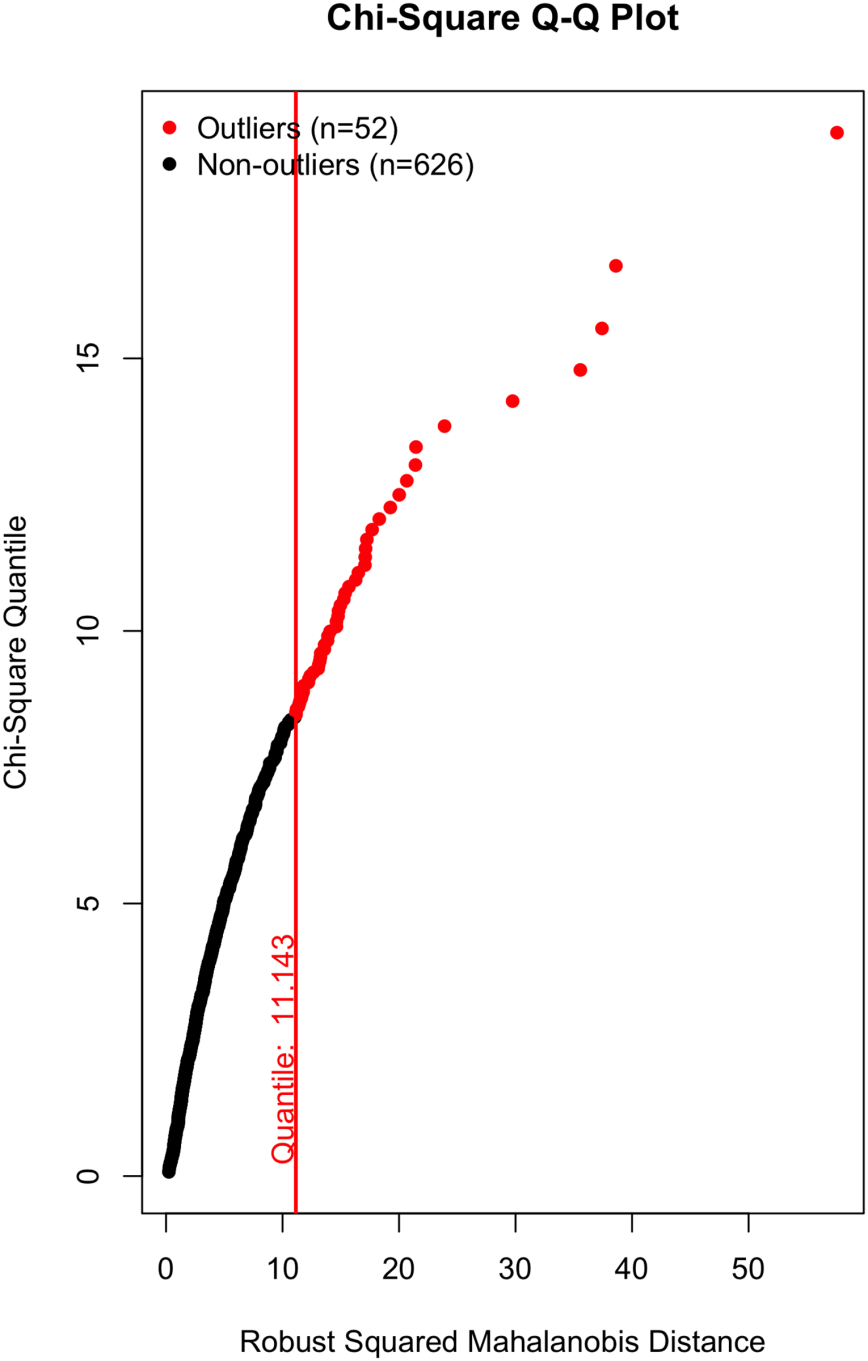
Multivariate outliers. Possession in Model 2.

**Fig 6:**
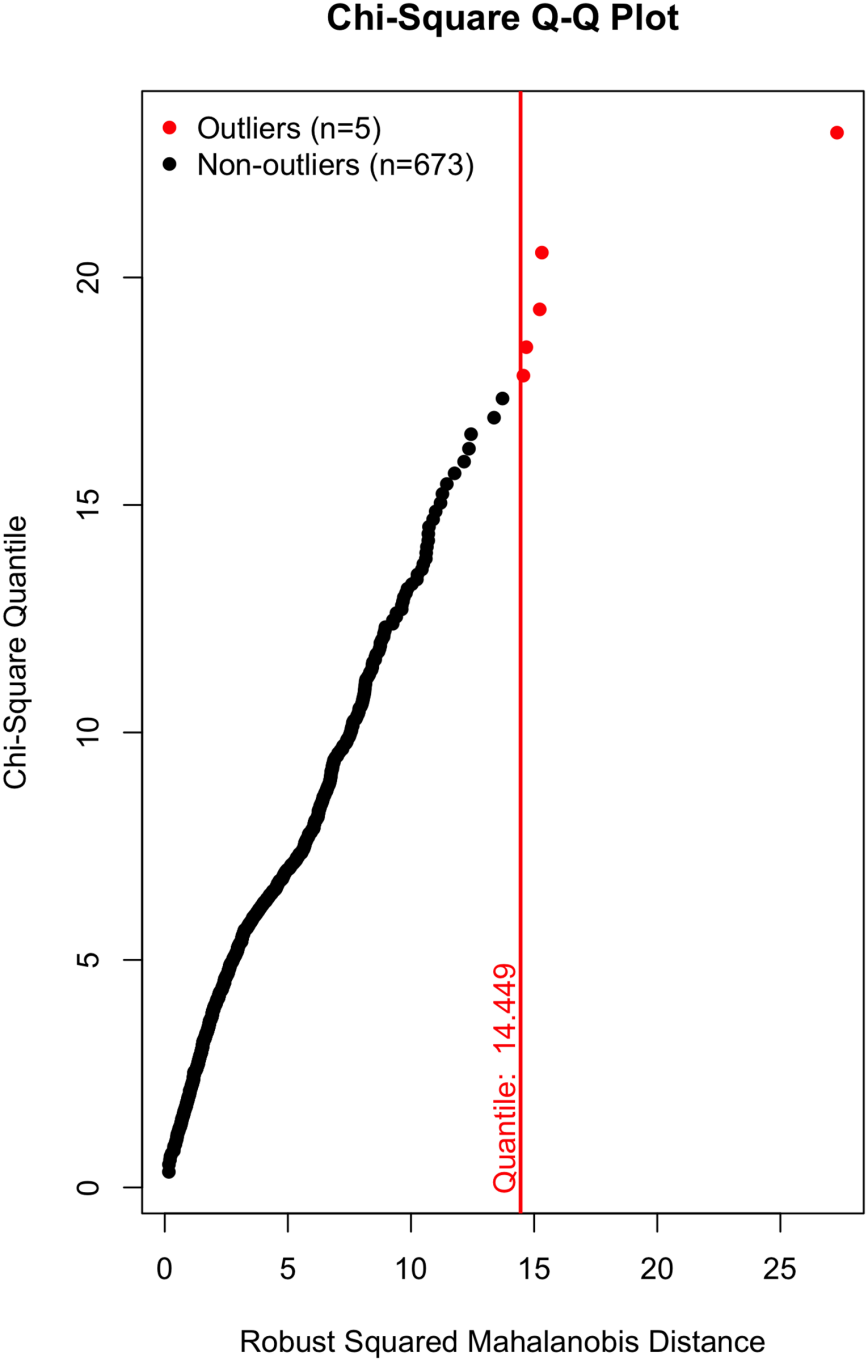
Multivariate outliers. Offense in Model 2.

**Fig 7:**
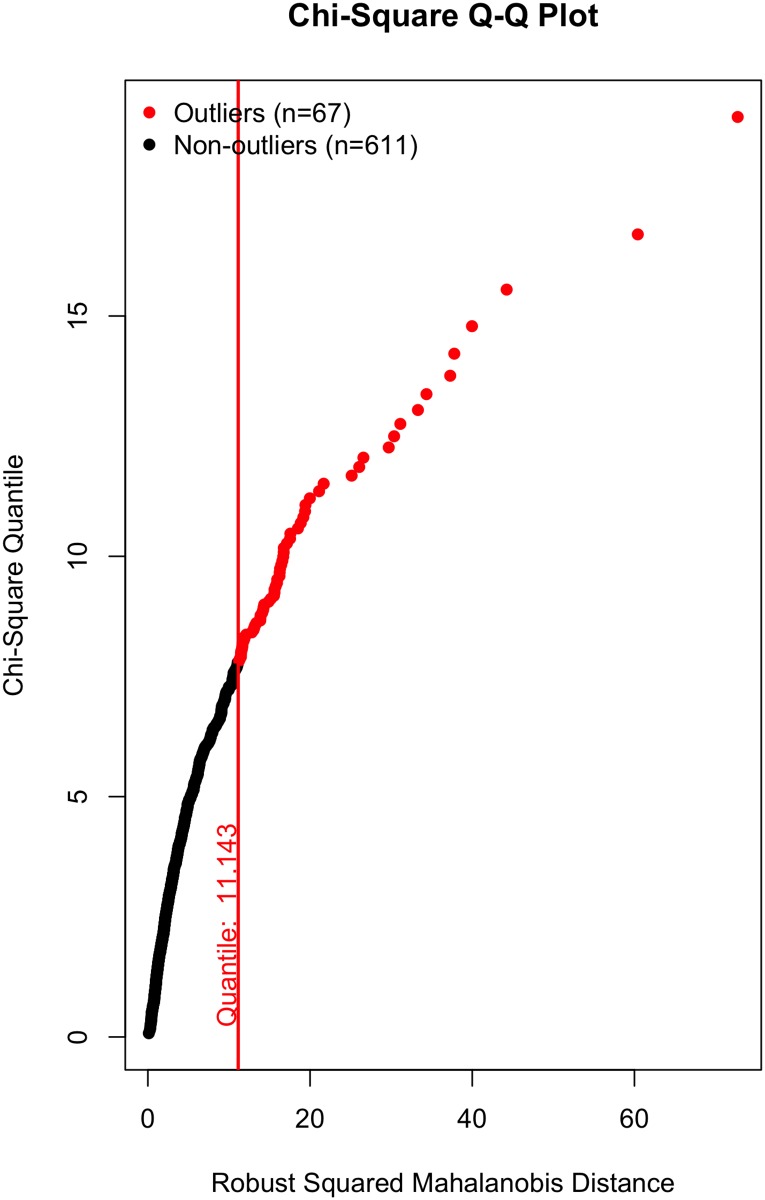
Multivariate outliers. Defense in Model 2.

All that said, a visual inspection of the data (Figs 2–7) suggests the violation of multivariate normality is due to a number of outliers. Although it is tempting to remove a subset of these outliers to establish multivariate normality [34], I contend there is no good theoretical reason to do so. On the one hand, a person could argue these outliers likely represent players who were “called up” from lower leagues to fill in for injured NHL players and should thus be removed; however, we (i) cannot reasonably conclude that from the data, and (ii) even if that were to be the case, these outliers still received substantial on-ice time and should thus be included in the data, even if they did not perform at an “NHL level”. Otherwise stated: we cannot exclude a player simply because they are an outlier, especially given all the evidence suggesting that normality violations in large samples produce unbiased parameter estimates (see above).

#### Identifiability

A model is of little use if its parameters do not have at least one unique solution (that is, there has to be at least one value for every unknown parameter, such as regression and factor weights), thus we need to make sure both the measurement and path models are identifiable [21]. As per MacDonald and Ho [21], identifiability of the measurement model was established by demonstrating independent clusters within the factor loadings. To achieve independent clusters, each latent variable had its raw metric loading fixed to 1.00 (in the case of *offense*, $P_{i}^{60}$ was arbitrarily chosen over $G_{f}^{60}$), and the model specified to not allow correlations between the residual variances of measured variables (Tables 5–8). Similarly, as per [21], identifiability of the path model was met by having (theoretically justified) uncorrelated disturbances between endogenous variables (referred to as the “orthogonality rule”).

**Table 5:** Factor structure of measurement model 1.

Latent Variable	Measured Variable	Unstandardized	Standardized	SE	Z-value	p-value
*Possession*						
	*C*_*p*_	1.00	0.77	-	-	-
	*τC*_*p*_	1.20	0.96	0.04	28.68	p <.01
	*δC*_*p*_	1.11	0.98	0.04	29.03	p <.01
*Offense*						
	$P_{i}^{60}$	1.00	0.63	-	-	-
	$G_{f}^{60}$	1.49	0.94	0.07	20.14	p <.01
	$\tau G_{f}^{60}$	1.89	0.96	0.09	20.50	p <.01
	$\delta G_{f}^{60}$	1.62	0.97	0.08	20.63	p <.01
*Defense*						
	$G_{a}^{60}$	1.00	0.90	-	-	-
	$\tau G_{a}^{60}$	1.21	0.95	0.03	43.15	p <.01
	$\delta G_{a}^{60}$	1.17	0.98	0.03	47.06	p <.01

**Table 6:** Model 1: Residual covariance between measured variables.

	*C*_*p*_	*τC*_*p*_	*δC*_*p*_	$P_{i}^{60}$	$G_{f}^{60}$	$\tau G_{f}^{60}$	$\delta G_{f}^{60}$	$G_{a}^{60}$	$\tau G_{a}^{60}$	$\delta G_{a}^{60}$
*C*_*p*_	5.88	-	-	-	-	-	-	-	-	-
*τC*_*p*_	0.00	1.14	-	-	-	-	-	-	-	-
*δC*_*p*_	0.00	0.00	0.47	-	-	-	-	-	-	-
$P_{i}^{60}$	0.00	0.00	0.00	0.19	-	-	-	-	-	-
$G_{f}^{60}$	0.00	0.00	0.00	0.00	0.04	-	-	-	-	-
$\tau G_{f}^{60}$	0.00	0.00	0.00	0.00	0.00	0.04	-	-	-	-
$\delta G_{f}^{60}$	0.00	0.00	0.00	0.00	0.00	0.00	0.02	-	-	-
$G_{a}^{60}$	0.00	0.00	0.00	0.00	0.00	0.00	0.00	0.04	-	-
$\tau G_{a}^{60}$	0.00	0.00	0.00	0.00	0.00	0.00	0.00	0.00	0.03	-
$\delta G_{a}^{60}$	0.00	0.00	0.00	0.00	0.00	0.00	0.00	0.00	0.00	0.01

**Table 7:** Factor structure of measurement model 2.

Latent Variable	Measured Variable	Unstandardized	Standardized	SE	Z-value	p-value
*Possession*						
	*C*_*p*_	1.00	0.77	-	-	-
	*OZFO*_*p*_	0.84	0.44	0.07	11.70	p <.01
	*τC*_*p*_	1.20	0.96	0.04	28.68	p <.01
	*δC*_*p*_	1.11	0.98	0.04	29.03	p <.01
*Offense*						
	$P_{i}^{60}$	1.00	0.63	-	-	-
	$G_{i}^{60}$	0.40	0.46	0.04	11.54	p <.01
	$A_{i}^{60}$	0.60	0.64	0.04	15.49	p <.01
	$G_{f}^{60}$	1.49	0.94	0.07	20.14	p <.01
	$\tau G_{f}^{60}$	1.89	0.96	0.09	20.50	p <.01
	$\delta G_{f}^{60}$	1.62	0.97	0.08	20.63	p <.01
*Defense*						
	$G_{a}^{60}$	1.00	0.90	-	-	-
	*DZFO*_*p*_	-1.12	-0.09	0.51	-2.21	p <.05
	$\tau G_{a}^{60}$	1.21	0.95	0.03	43.15	p <.01
	$\delta G_{a}^{60}$	1.17	0.98	0.03	47.06	p <.01

**Table 8:** Model 2: Residual covariance between measured variables.

	*C*_*p*_	*OZFO*_*p*_	*τC*_*p*_	*δC*_*p*_	$P_{i}^{60}$	$G_{i}^{60}$	$A_{i}^{60}$	$G_{f}^{60}$	$\tau G_{f}^{60}$	$\delta G_{f}^{60}$	$G_{a}^{60}$	*DZFO*_*p*_	$\tau G_{a}^{60}$	$\delta G_{a}^{60}$
*C*_*p*_	5.85	-	-	-	-	-	-	-	-	-	-	-	-	-
*OZFO*_*p*_	0.00	24.59	-	-	-	-	-	-	-	-	-	-	-	-
*τC*_*p*_	0.00	0.00	0.94	-	-	-	-	-	-	-	-	-	-	-
*δC*_*p*_	0.00	0.00	0.00	0.65	-	-	-	-	-	-	-	-	-	-
$P_{i}^{60}$	0.00	0.00	0.00	0.00	0.18	-	-	-	-	-	-	-	-	-
$G_{i}^{60}$	0.00	0.00	0.00	0.00	0.00	0.08	-	-	-	-	-	-	-	-
$A_{i}^{60}$	0.00	0.00	0.00	0.00	0.00	0.00	0.07	-	-	-	-	-	-	-
$G_{f}^{60}$	0.00	0.00	0.00	0.00	0.00	0.00	0.00	0.04	-	-	-	-	-	-
$\tau G_{f}^{60}$	0.00	0.00	0.00	0.00	0.00	0.00	0.00	0.00	0.04	-	-	-	-	-
$\delta G_{f}^{60}$	0.00	0.00	0.00	0.00	0.00	0.00	0.00	0.00	0.00	0.02	-	-	-	-
$G_{a}^{60}$	0.00	0.00	0.00	0.00	0.00	0.00	0.00	0.00	0.00	0.00	0.04	-	-	-
*DZFO*_*p*_	0.00	0.00	0.00	0.00	0.00	0.00	0.00	0.00	0.00	0.00	0.00	29.09	-	-
$\tau G_{a}^{60}$	0.00	0.00	0.00	0.00	0.00	0.00	0.00	0.00	0.00	0.00	0.00	0.00	0.03	-
$\delta G_{a}^{60}$	0.00	0.00	0.00	0.00	0.00	0.00	0.00	0.00	0.00	0.00	0.00	0.00	0.00	0.01

### Testing the models

I first performed a visual inspection of the correlations between all relevant metrics (Table 9). As expected, correlations between metrics comprising latent variables were moderate to strong, correlations between metrics comprising *possession* and *offense* were moderate, with weak to absent correlations everywhere else, the exception being *DZFO*_*p*_, which exhibited a (mostly) moderate negative correlation with all the measured variables.

**Table 9:** Correlations between measured variables.

	*C*_*p*_	*OZFO*_*p*_	$\tau C_{p}^{60}$	$\delta C_{p}^{60}$	$P_{i}^{60}$	$G_{i}^{60}$	$A_{i}^{60}$	$G_{f}^{60}$	$\tau G_{f}^{60}$	$\delta G_{f}^{60}$	$G_{a}^{60}$	*DZFO*_*p*_	$\tau G_{a}^{60}$	$\delta G_{a}^{60}$
*C*_*p*_	1.00	-	-	-	-	-	-	-	-	-	-	-	-	-
*OZFO*_*p*_	0.46	1.00	-	-	-	-	-	-	-	-	-	-	-	-
$\tau C_{p}^{60}$	0.73	0.45	1.00	-	-	-	-	-	-	-	-	-	-	-
$\delta C_{p}^{60}$	0.75	0.40	0.94	1.00	-	-	-	-	-	-	-	-	-	-
$P_{i}^{60}$	0.30	0.26	0.35	0.34	1.00	-	-	-	-	-	-	-	-	-
$G_{i}^{60}$	0.24	0.21	0.26	0.27	0.83	1.00	-	-	-	-	-	-	-	-
$A_{i}^{60}$	0.27	0.23	0.33	0.31	0.86	0.43	1.00	-	-	-	-	-	-	-
$G_{f}^{60}$	0.35	0.37	0.40	0.36	0.63	0.44	0.63	1.00	-	-	-	-	-	-
$\tau G_{f}^{60}$	0.33	0.39	0.45	0.39	0.60	0.41	0.59	0.90	1.00	-	-	-	-	-
$\delta G_{f}^{60}$	0.31	0.35	0.40	0.37	0.61	0.43	0.59	0.91	0.94	1.00	-	-	-	-
$G_{a}^{60}$	-0.13	0.05	-0.04	-0.07	0.03	0.04	0.01	0.07	0.10	0.08	1.00	-	-	-
*DZFO*_*p*_	-0.43	-0.86	-0.43	-0.40	-0.25	-0.22	-0.21	-0.35	-0.35	-0.32	-0.06	1.00	-	-
$\tau G_{a}^{60}$	-0.06	0.10	-0.06	-0.09	0.05	0.06	0.03	0.10	0.11	0.07	0.85	-0.09	1.00	-
$\delta G_{a}^{60}$	-0.06	0.08	-0.06	-0.10	0.03	0.04	0.01	0.07	0.07	0.06	0.88	-0.08	0.93	1.00

Looking at the standardized (*β*) and unstandardized (B) regression weights in Table 10, we see that, for both measurement models, *possession* was negatively related to *defense* and positively related to *offense*, and that *offense* was positively related to *defense*. Moreover, with both measurement models, the effect between *possession* and *defense* was weakened when *offense* was introduced as a mediator (Model 1: *β* = −0.09 vs *β* = −0.15, Model 2: *β* = −0.08 vs *β* = −0.14; Tables 10 & 11), with *offense* exhibiting an indirect effect of *β* = 0.06 under both models (Table 11). Thus, *offense* appears to act as a partial mediator between *possession* and *defense* (using Sobel’s method for p- and Z-values [35]).

**Table 10:** Direct effects for each measurement model.

			B	*β*	SE	Z-value	p-value
Model 1							
	*defense*						
		*possession*	-0.02	-0.15	0.01	-3.34	p <.01
		*offense*	0.17	0.14	0.05	3.21	p <.01
	*offense*						
		*possession*	0.05	0.42	0.01	9.70	p <.01
Model 2							
	*defense*						
		*possession*	-0.02	-0.14	0.01	-3.20	p <.01
		*offense*	0.16	0.14	0.05	3.20	p <.01
	*offense*						
		*possession*	0.06	0.43	0.01	10.10	p <.01

**Table 11:** Mediating effects of *offense* on the relationship between *possession* and *defense*.

	Effect	B	*β*	SE	Z-value	p-value
Model 1						
	Total	-0.01	-0.09	0.01	-2.20	p <.05
	Indirect	0.01	0.06	0.003	3.11	p <.01
Model 2						
	Total	-0.01	-0.08	0.01	-2.01	p <.05
	Indirect	0.01	0.06	0.003	3.10	p <.01

Assessments of model fit can be seen in Table 12. As expected given our large sample size, a significant *χ*^2^ was observed. However, in line with [21, 36], the *χ*^2^ metric was ignored in favor of the standardized root mean square residual (SRMR), the Tucker-Lewis index (TLI), and comparative fit index (CFI). The SRMR was selected as the indicator of absolute model fit, and is simply the standardize difference between observed and predicted correlations (an SRMR of zero implies perfect fit, with anything above.05 being a poor fit) [36]. For a measure of fit relative to the baseline model (where all measured variables are uncorrelated) I selected the TLI with a cutoff value of .95 [36]. However, because the TLI is a centrality based measure, the CFI (using a .95 cutoff) was also included [36].

**Table 12:** Assessments of model fit.

	Fit Index	Value	Good/Bad
Model 1			
	*χ*^2^	232	Bad
	SRMR	0.03	Good
	TLI	0.96	Good
	CFI	0.97	Good
Model 2			
	*χ*^2^	13582	Bad
	SRMR	0.16	Bad
	TLI	0.23	Bad
	CFI	0.38	Bad

Overall, model 2 proved to be a poor fit, with model 1 being a good fit. To test whether model 2’s poor fit was due to the large residual covariances for *OZFO*_*p*_ and *DZFO*_*p*_ (see Table 8), those variables were removed from the measurement model and the full SEM tested again. However, this third model proved a similarly poor fit (SRMR = 0.09, TLI = 0.21, CFI = 0.39). Further, an examination of the standardized residual covariances of each fitted model (Tables 13 & 14) shows that the estimates generated by model 1 more closely match the observations in the data than the estimates generated by model 2. For example, model 1 had residual covariances of ±3 for three of the 55 values (3.38, −4.20, −5.31), whereas model 2 had ±3 for 26 of the 105 values (4.79, −10.38, 3.76, 3.34, 5.54, 6.17, 5.03, −17.01, 3.77, 3.19, 3.50, −10.44, −3.96, −5.87, −9.92, 13.49, 12.12, −6.24, 4.57, −9.14, −6.38, −5.49, −5.14, −8.52, −8.53, −7.87), which constitutes 5.46% and 24.76% of all possible values, respectively. That said, the preponderance of the ±3 values in model 2 come from *OZFO*_*p*_ and *DZFO*_*p*_; however, as noted earlier, the removal of these two measured variables did not produce a good model fit.

**Table 13:** Model 1: Residual covariances of fitted model (implied versus observed).

	*C*_*p*_	$\tau C_{p}^{60}$	$\delta C_{p}^{60}$	$P_{i}^{60}$	$G_{f}^{60}$	$\tau G_{f}^{60}$	$\delta G_{f}^{60}$	$G_{a}^{60}$	$\tau G_{a}^{60}$	$\delta G_{a}^{60}$
*C*_*p*_	0.00/NA	NA	1.22	3.03	1.77	0.86	-0.35	-2.47	0.112	0.38
$\tau C_{p}^{60}$	-0.12	0.00/NA	0.14	3.38	1.67	4.31	1.19	1.91	1.49	1.73
$\delta C_{p}^{60}$	0.05	0.01	0.00/NA	2.94	-2.72	-1.03	-5.31	0.41	-1.04	-1.78
$P_{i}^{60}$	0.21	0.20	0.15	0.00/NA	3.09	-4.20	-2.59	-0.37	0.12	-0.75
$G_{f}^{60}$	0.09	0.05	-0.05	0.01	0.00/NA	NA	NA	-0.07	1.54	-0.15
$\tau G_{f}^{60}$	0.05	0.15	-0.02	-0.01	-0.00	0.00/NA	0.69	1.51	2.74	-0.17
$\delta G_{f}^{60}$	-0.02	0.03	-0.05	-0.00	+0.00	+0.00	0.00/NA	0.43	0.14	-2.21
$G_{a}^{60}$	-0.12	0.06	0.01	-0.00	+0.00	0.01	+0.00	0.00/0.00	NA	0.29
$\tau G_{a}^{60}$	0.01	0.04	-0.02	+0.00	0.01	0.01	+0.00	+0.00	0.00/0.00	+0.00
$\delta G_{a}^{60}$	0.02	0.03	-0.02	-0.01	-0.00	-0.00	-0.01	+0.00	+0.00	0.00/0.00

Note: Lower triangle contains unstandardized residuals. Upper triangle contains standardized residuals. 0.00 refers to true zeros. +0.00 and −0.00 refer to rounded zeros. Along the diagonal, the first term refers to the unstandardized residual, and the second term the standardized residual.

**Table 14:** Model 2: Residual covariances of fitted model (implied versus observed).

	*C*_*p*_	*OZFO*_*p*_	$\tau C_{p}^{60}$	$\delta C_{p}^{60}$	$P_{i}^{60}$	$G_{i}^{60}$	$A_{i}^{60}$	$G_{f}^{60}$	$\tau G_{f}^{60}$	$\delta G_{f}^{60}$	$G_{a}^{60}$	*DZFO*_*p*_	$\tau G_{a}^{60}$	$\delta G_{a}^{60}$
*C*_*p*_	0.00/0.04	4.79	NA	1.85	2.61	2.44	1.80	1.31	0.40	-0.83	-2.66	-10.38	-0.07	1.66
*OZFO*_*p*_	2.44	0.00/NA	1.91	NA	3.76	3.34	2.95	5.54	6.17	5.03	2.21	-17.01	3.77	3.19
$\tau C_{p}^{60}$	-0.22	0.30	0.00/NA	0.54	2.78	2.19	2.19	0.50	3.50	-0.35	1.65	-10.44	1.15	1.30
$\delta C_{p}^{60}$	0.11	-0.58	0.01	0.00/NA	2.42	2.33	1.45	-3.96	-2.07	-5.87	-0.02	-9.92	-1.54	-2.37
$P_{i}^{60}$	0.17	0.42	0.16	0.12	0.00/NA	13.49	12.12	1.37	NA	NA	-0.44	-6.24	0.06	-0.84
$G_{i}^{60}$	1.00	0.22	0.08	0.08	0.09	0.00/0.00	4.57	-0.11	-9.14	-6.38	0.33	-5.49	0.65	0.06
$A_{i}^{60}$	0.07	0.20	0.08	0.05	0.08	0.01	0.00/NA	2.12	NA	NA	-1.05	-5.14	-0.60	-1.40
$G_{f}^{60}$	0.07	0.58	0.01	-0.07	+0.00	+0.00	+0.00	0.00/NA	NA	0.19	-0.10	-8.52	1.53	-0.20
$\tau G_{f}^{60}$	0.02	0.81	0.11	-0.04	-0.01	-0.01	-0.01	-0.00	0.00/NA	1.54	1.49	-8.53	2.70	-0.18
$\delta G_{f}^{60}$	-0.04	0.55	-0.01	-0.07	-0.01	-0.00	-0.01	+0.00	+0.00	0.00/NA	0.42	-7.87	0.13	-2.08
$G_{a}^{60}$	-0.13	0.20	0.05	-0.00	-0.00	+0.00	-0.01	+0.00	0.01	+0.00	0.00/NA	1.10	NA	0.33
*DZFO*_*p*_	-8.84	-25.72	-8.56	-7.27	-0.75	-0.37	-0.38	-1.05	-1.30	-1.01	0.04	+0.00/0.11	-1.23	0.15
$\tau G_{a}^{60}$	-0.00	0.39	0.03	-0.03	+0.00	+0.00	-0.00	0.01	0.01	+0.00	+0.00	-0.04	0.00/0.00	NA
$\delta G_{a}^{60}$	-0.01	0.30	0.02	-0.03	-0.01	+0.00	-0.01	-0.00	-0.00	-0.01	+0.00	+0.00	+0.00	0.00/0.00

Note: Lower triangle contains unstandardized residuals. Upper triangle contains standardized residuals. 0.00 refers to true zeros. +0.00 and −0.00 refer to rounded zeros. Along the diagonal, the first term refers to the unstandardized residual, and the second term the standardized residual.

To assess model 1’s ability to generalize beyond the data it was fitted on, the parameter estimates generated by the model were applied to a new set of data drawn from puckalytics for the 2015/2016 NHL season, once again using lavaan [27]. These parameter estimates calculated predicted factor scores for latent variables, which were then used to compute predicted values for measured variables; the error between these predicted values and the values observed in the data were then compared (Table 15). I elected to use the mean absolute error (MAE) to get an unweighted indication of accuracy, as well as the root mean square error (RMSE) to penalize large errors.

**Table 15:** Comparing predicted and observed values for measured variables (2015/2016 season).

Latent Variable	Measured Variable	Observed *M* (*SD*)	MAE	RMSE	*r*
*Possession*					
	*C*_*p*_	49.41 (4.06)	2.01	2.38	0.81
	*τC*_*p*_	-0.59 (3.98)	0.72	1.01	0.97
	*δC*_*p*_	-0.27 (3.54)	0.33	0.46	0.99
*Offense*					
	$P_{i}^{60}$	1.09 (0.59)	0.37	0.45	0.65
	$G_{f}^{60}$	2.05 (0.61)	0.15	0.18	0.96
	$\tau G_{f}^{60}$	-0.12 (0.74)	0.12	0.17	0.97
	$\delta G_{f}^{60}$	-0.06 (0.63)	0.09	0.11	0.97
*Defense*					
	$G_{a}^{60}$	2.15 (0.50)	0.16	0.20	0.92
	$\tau G_{a}^{60}$	-0.01 (0.58)	0.12	0.18	0.95
	$\delta G_{a}^{60}$	-0.03 (0.53)	0.05	0.07	0.99

Here, the model provided accurate *τC*_*p*_ and *δC*_*p*_ predictions, with MAEs/RMSEs of 0.72/1.01 and 0.33/0.46, respectively. That said, the MAE for *C*_*p*_ predictions was 2.01 (RMSE = 2.38), which is approximately one half of a standard deviation in observed *C*_*p*_ scores.

With respect to *offense*, MAE and RMSE values for $G_{f}^{60}$, $\tau G_{f}^{60}$, and $\delta G_{f}^{60}$ suggest a high level of accuracy in the model’s predictions, but $P_{i}^{60}$ predictions were less accurate, with a MAE roughly 63% of a standard deviation in observed $P_{i}^{60}$ scores.

*Defense* indicators followed a similar prediction pattern as *possession* and *offense* indicators, with $G_{a}^{60}$ seeing the greatest prediction error; however, it is of particular note that goals against metrics were predicted with nearly the exact same accuracy as goals for metrics. It should also be noted that although the greatest prediction errors involve measured variables whose factor loadings were fixed to 1 while fitting the SEM, this is merely a coincidence, and selecting different variables to fix at 1 does not alter predictions. The most probable explanation for why these variables generate the worst predictions is that raw metrics exhibit the most year-over-year variability, which makes them the hardest to predict. Given that all of the correlations between predicted values and observed values were strong, and that only $P_{i}^{60}$ had a MAE greater than one half of a standard deviation in observed scores (with most falling substantially below that), it is reasonable to conclude that model 1’s parameter estimates generalize beyond the data they were derived from.

Finally, although correlations between predicted values and observed values were high across all measured variables, $P_{i}^{60}$’s correlation was noticeably weaker than the rest.

To examine the stability of performance predictions across a longer time-period, parameter estimates from model 1 were used to generate predicted values for the 2010/2011 and 2016/2017 NHL seasons (drawn from puckalytics; Table 16), which are both one full season removed from the data our model was fitted on (2012/13–2014/2015), and have five full seasons in-between them, which is approximately the length of an average NHL career [37].

**Table 16:** Comparing predicted and observed values for measured variables (2010/2011 & 2016/2017 seasons).

Latent Variable	Measured Variable	Observed *M* (*SD*)	MAE	RMSE	*r*
*Possession*					
	2010/2011 *C*_*p*_	49.56 (4.19)	1.94	2.37	0.83
	2016/2017 *C*_*p*_	49.61 (3.67)	1.55	1.99	0.84
	2010/2011 *τC*_*p*_	-0.34 (4.11)	0.69	0.94	0.97
	2016/2017 *τC*_*p*_	-0.39 (3.92)	0.69	0.97	0.97
	2010/2011 *δC*_*p*_	-0.10 (3.63)	0.33	0.44	0.99
	2016/2017 *δC*_*p*_	-0.07 (3.45)	0.33	0.46	0.99
*Offense*					
	2010/2011 $P_{i}^{60}$	1.21 (0.64)	0.44	0.52	0.61
	2016/2017 $P_{i}^{60}$	1.17 (0.58)	0.37	0.45	0.63
	2010/2011 $G_{f}^{60}$	2.20 (0.63)	0.19	0.23	0.94
	2016/2017 $G_{f}^{60}$	2.17 (0.60)	0.21	0.25	0.91
	2010/2011 $\tau G_{f}^{60}$	-0.08 (0.74)	0.14	0.19	0.97
	2016/2017 $\tau G_{f}^{60}$	-0.08 (0.67)	0.13	0.18	0.97
	2010/2011 $\delta G_{f}^{60}$	-0.03 (0.64)	0.09	0.12	0.98
	2016/2017 $\delta G_{f}^{60}$	-0.03 (0.57)	0.09	0.12	0.98
*Defense*					
	2010/2011 $G_{a}^{60}$	2.28 (0.53)	0.20	0.25	0.92
	2016/2017 $G_{a}^{60}$	2.22 (0.51)	0.18	0.23	0.91
	2010/2011 $\tau G_{a}^{60}$	-0.03 (0.59)	0.12	0.18	0.96
	2016/2017 $\tau G_{a}^{60}$	-0.04 (0.58)	0.12	0.17	0.96
	2010/2011 $\delta G_{a}^{60}$	-0.04 (0.55)	0.05	0.07	0.99
	2016/2017 $\delta G_{a}^{60}$	-0.05 (0.53)	0.05	0.07	0.99

Once again, a similar pattern emerged whereby *C*_*p*_, $P_{i}^{60}$, and $G_{a}^{60}$ all suffered from the least accurate predictions. However, the observed means, observed standard deviations, and accuracy of predictions proved to be highly similar between the two seasons (as well as the 2015/2016 season), and it is reasonable to conclude that model 1’s parameter estimates generalize across longer time-periods.

It is important to stress, however, that the method of prediction used in the above analyses is conceptually different from “regression-like” prediction; instead of using the known values of independent variables to predict scores on some unknown dependent variable, we are using the known parameter estimates of the fitted model to predict values for all of the measured variables. That is, we are not predicting how a player will perform in the future, we are examining whether model 1’s parameter estimates can accurately predict measured variables in another dataset; if the parameter estimates do not provide accurate predictions, then the parameter estimates do not generalize beyond the data they were derived from. That said, should the need arise, we can perform “regression-like” prediction by regressing latent variable factor scores onto whatever measured variable(s) we want to predict. (Because a latent variable is simply an abstract concept that exists as the combination of relevant measured variables, unless there are good theoretical reasons to do otherwise, predictions about future performance are (likely) best made with the measured variables themselves).

### Ranking player performance

Just as a person’s intelligence is comprised of scores on various abstract concepts (e.g., working memory, verbal reasoning, etc.), which are themselves comprised of scores on a variety of measured variables, so-to is a hockey player’s overall performance. That is, a player’s overall performance is simply a composition of their scores on latent variables. To this end, latent variable factor scores were obtained for players who played at least half of the 2016/2017 season (41 games), and were combined to generate *overall* performance scores.

With respect to *possession* (Table 17), four of the five top-ranking players are what would be considered “elite” forwards, with the other player (Andrew Cogliano) being a “utility” forward. Moreover, defensemen were underrepresented in the top 20, filling only 20% of the spots despite comprising roughly 33% of each team’s skaters in any given game.

**Table 17:** Top 20 players based on *possession* scores (2016/2017).

Player	Position	GP	TOI	*Possession*
Patrice Bergeron	F	79	1035	7.96
Artemi Panarin	F	82	1258	7.92
Andrew Cogliano	F	82	1063	7.22
Brad Marchand	F	80	1097	7.15
John Tavares	F	77	1175	7.10
Michal Kempny	D	50	699	6.69
Blake Wheeler	F	82	1188	6.68
Nino Niederreiter	F	82	1041	6.66
Chris Kreider	F	75	1036	6.59
Colin Miller	D	61	805	6.47
Dougie Hamilton	D	81	1267	6.46
Ryan Johansen	F	82	1146	6.17
Michael Frolik	F	82	1071	6.16
Matthew Tkachuk	F	76	930	6.15
Beau Bennett	F	65	764	6.11
Jaromir Jagr	F	82	1129	6.10
Taylor Hall	F	72	1094	6.05
Mark Stone	F	71	1015	6.01
Derick Brassard	F	81	1113	5.92
Brayden McNabb	D	49	701	5.51

Note: *M* = 0.11, Range: 7.96 to −9.29.

*Offense* scores (Table 18) identified Connor McDavid and Brent Burns as the highest ranked forward and defensemen, respectively. However, there are some notable names outside the top 20; Sidney Crosby ranked 63rd (0.42), Patrick Kane 73rd (0.38), and Alexander Ovechkin 78th (0.36). All three of these players are excellent talents whom are consistently some of the leagues top scorers, but because *offense*, as an abstract concept, is much more than raw point production, the rankings produced by model 1 and the rankings based on year-end point totals will, and should, be different. For example, David Krejci and Vincent Trocheck are both centermen who played in all 82 games and scored 54 points (23 goals and 31 assists), yet Trocheck had an *offense* score of 0.23, whereas Krejci had a score of 0.11. This is largely because Trocheck managed to generate the same output while playing on a substantially worse team.

**Table 18:** Top 20 players based on *offense* scores (2016/2017).

Player	Position	GP	TOI	*Offense*
Connor McDavid	F	82	1309	0.92
Conor Sheary	F	61	836	0.87
Mike Hoffman	F	74	1001	0.84
Jason Zucker	F	79	1104	0.80
Viktor Arvidsson	F	80	1064	0.80
Jeff Skinner	F	78	1139	0.78
Mark Scheifele	F	79	1217	0.77
Patrik Laine	F	73	1031	0.76
Brad Marchand	F	80	1097	0.76
Jannik Hansen	F	42	583	0.74
Brent Burns	D	82	1519	0.73
Evgeni Malkin	F	62	871	0.71
Nikita Kucherov	F	74	1097	0.70
Pavel Buchnevich	F	41	451	0.69
Thomas Vanek	F	68	759	0.68
Tyler Bozak	F	78	1037	0.68
Sean Couturier	F	66	921	0.66
Matthew Tkachuk	F	76	930	0.64
Henrik Zetterberg	F	82	1274	0.62
Colin Wilson	F	70	872	0.61

Note: *M* = 0.03, Range: 0.92 to −0.93.

Because *defense* scores reflect goals against, smaller values indicate better performance (Table 19). Here, *defense* rankings were notably absent of what would be considered “elite” offensive talents, be they forwards (e.g., Connor McDavid) or defensemen (e.g., Brent Burns). Instead, the rankings primarily consist of “utility” forwards and defensemen, which is to be expected given the inverse relationship between *offense* and *defense*.

**Table 19:** Top 20 players based on *defense* scores (2016/2017).

Player	Position	GP	TOI	*Defense*
Anton Slepyshev	F	41	447	-1.10
John Mitchell	F	65	745	-0.98
Stefan Noesen	F	44	458	-0.95
Colton Sissons	F	58	578	-0.87
Matt Read	F	63	745	-0.87
Brett Richie	F	78	871	-0.84
Shea Weber	D	78	1388	-0.83
Andrew Copp	F	64	667	-0.81
Shane Doan	F	74	965	-0.80
Auston Watson	F	77	786	-0.79
Pierre-Edouard Bellemare	F	82	797	-0.77
Kyle Clifford	F	73	768	-0.76
Tyler Graovac	F	52	504	-0.75
Jayson Megna	F	58	662	-0.73
Mark Giordano	D	81	1333	-0.73
Jaccob Slavin	D	81	1482	-0.70
Matt Martin	F	82	699	-0.69
Kyle Palmieri	F	80	1036	-0.68
Brendan Perlini	F	57	723	-0.68
Chris Wideman	D	76	906	-0.68

Note: *M* = 0.01, Range: 1.42 to −1.10.

As stated earlier, a player’s *overall* score exists as some combination of *possession*, *offense*, and *defense*; how these three scores are combined, however, depends on how much emphasis a person places in each of the above factors; if a person believes *offense* is more important than *defense*, then they will assign more weight to those scores. Regardless of how this emphasis is distributed, we must scale *possession* scores down by a factor of 10. This is because *possession* factor scores take into consideration *C*_*p*_ scores, which are an order of magnitude larger than all other measured variables, thus leading to *possession* scores being an order of magnitude larger than *offense* and *defense* scores. If we do not perform this re-scaling, then *overall* scores will be almost entirely determined by *possession* scores. Moreover, because smaller *defense* scores indicate superior performance, *defense* scores should be subtracted, not added, to *possession* and *offense* scores. Otherwise stated, to generate *overall* scores, we decided if, and by how much, we want to weigh each of the latent variables, then compute *overall* scores by scaling *possession* scores down by a factor of 10, adding that value to *offense* scores, then subtracting *defense* scores from that new value.

To evaluate *overall* rankings, two formulations were constructed (Tables 20 & 21):

Overall=Possession/10+Offense−Defense

$$\begin{array}{c} Overall=Possession/10+2\left(Offense\right)-0.5\left(Defense\right) \end{array}$$

and the top 20 players identified.

**Table 20:** Top 20 players based on *overall* scores: Unweighted (2016/2017).

Player	Position	GP	TOI	*Possession*	*Offense*	*Defense*	*Overall*
Matthew Tkachuk	F	76	930	6.15	0.64	-0.34	1.60
Henrik Zetterberg	F	82	1274	3.03	0.62	-0.63	1.56
Mark Giordano	D	81	1333	3.88	0.41	-0.73	1.53
Anthony Mantha	F	60	797	5.32	0.50	-0.49	1.52
Nikita Kucherov	F	74	1097	5.17	0.70	-0.25	1.47
Aleksander Barkov	F	61	849	4.34	0.57	-0.42	1.43
Jaromir Jagr	F	82	1129	6.10	0.49	-0.32	1.42
Jason Zucker	F	79	1104	1.31	0.80	-0.46	1.39
Jaccob Slavin	D	81	1482	2.16	0.46	-0.70	1.38
Artemi Panarin	F	82	1258	7.92	0.55	0.01	1.34
Stefan Noesen	F	44	458	1.96	0.19	-0.95	1.33
Mike Hoffman	F	74	1001	1.97	0.84	-0.29	1.33
Brandon Saad	F	82	1137	5.40	0.30	-0.49	1.33
Dougie Hamilton	D	81	1267	6.46	0.45	-0.19	1.29
Patrice Bergeron	F	79	1035	7.96	0.10	-0.39	1.28
Mark Stone	F	71	1015	6.01	0.56	-0.10	1.27
Nino Niederrieter	F	82	1041	6.66	0.47	-0.12	1.26
Conor Sheary	F	61	836	2.25	0.87	-0.15	1.25
Brent Burns	D	82	1519	4.05	0.73	-0.11	1.25
Connor McDavid	F	82	1309	3.49	0.92	0.04	1.22

Note: *Overall* = *Possession*/10 + *Offense* − *Defense*, *M* = 0.04, Range: 1.60 to −1.77. Scores rounded to two decimal places.

**Table 21:** Top 20 players based on *overall* scores: *Offense* focused (2016/2017).

Player	Position	GP	TOI	*Possession*	*Offense*	*Defense*	*Overall*
Connor McDavid	F	82	1309	3.49	0.92	0.04	2.16
Brad Marchand	F	80	1097	7.15	0.76	0.28	2.09
Matthew Tkachuk	F	76	930	6.15	0.64	-0.34	2.07
Conor Sheary	F	61	836	2.25	0.87	-0.15	2.05
Nikita Kucherov	F	74	1097	5.17	0.70	-0.25	2.04
Mike Hoffman	F	74	1001	1.97	0.84	-0.29	2.03
Jason Zucker	F	79	1104	1.31	0.80	-0.46	1.97
Brent Burns	D	82	1519	4.05	0.73	-0.11	1.92
Artemi Panarin	F	82	1258	7.92	0.55	0.01	1.89
Henrik Zetterberg	F	82	1274	3.03	0.62	-0.63	1.86
Viktor Arvidsson	F	80	1064	4.58	0.80	0.44	1.84
Aleksander Barkov	F	61	849	4.34	0.57	-0.42	1.79
Mark Stone	F	71	1015	6.01	0.56	-0.10	1.78
Anthony Mantha	F	60	797	5.32	0.50	-0.49	1.78
Jaromer Jagr	F	82	1129	6.10	0.49	-0.32	1.76
Evgeni Malkin	F	62	871	2.40	0.71	-0.08	1.70
Sean Couturier	F	66	921	4.16	0.66	0.10	1.69
Nino Niederreiter	F	82	1041	6.66	0.47	-0.12	1.68
Dougie Hamilton	D	81	1267	6.46	0.45	-0.19	1.65
Mark Giordano	D	81	1333	3.88	0.41	-0.73	1.57

Note: *Overall* = *Possession*/10 + 2(*Offense*) − 0.5(*Defense*), *M* = 0.08, Range: 2.16 to −2.61. Scores rounded to two decimal places.

When all three latent variables were unweighted, the most interesting name on the list was Stephan Noesen, a rookie who spent the three seasons prior in the American Hockey League, and who owes his spot in the top 20 to an excellent *defense* score and an above average *possession* score. Fellow rookie Matthew Tkachuk topped the list, with league’s leading scorer, Connor McDavid, coming in 20th due to a below average *defense* score. Of course, whether one believes Matthew Tkachuk outperforms Connor McDavid depends on whether one gives equal weighting to *possession*, *offense*, and *defense*. When *offense* is given greater importance and *defense* less importance, a different picture emerges.

Given how much harder it is to score goals than prevent them, this *offense* focused weighting (arguably) gives a more accurate depiction of player performance. Here, Connor McDavid, the leagues leading scorer and Hart Trophy winner (awarded to the league’s most valuable player), tops the list, with Mathew Tkachuk falling back two spots to number three. Moreover, the list is comprised largely of forwards, with the top defenseman being Brent Burns, the James Norris Memorial Trophy winner (awarded to the league’s best defenseman).

## Conclusion

Paralleling Thomas and colleagues’ [10] work demonstrating that the interactive effects between players impacts individual performance, my findings suggest that *offense* mediates the relationship between *possession* and *defense*, and that this mediation occurs under multiple measurement models. One possible explanation for this relationship is that players who score lots of points are more likely to “cheat” for offense than their low scoring counterparts, which leads them to neglect defensive responsibilities that would otherwise have prevented goal(s) against. This theory is tangentially supported by zone entry research suggesting that controlled entries into the offensive zone produce more goals than attacking after the puck has been shot into the offensive zone, and that controlled zone entries are thought to be a higher risk play as a turn-over at the offensive blueline can often lead to a dangerous scoring chance against [38]. Thus, it may be the case that those who attempt to maintain possession as they enter the offensive zone—as opposed to choosing the safer option of simply shooting the puck in—not only produce more shots and goal for, but also more high-risk turnovers, which, subsequently, leads to more goals against.

Another possible explanation rests in the idea that scoring points at the NHL level is incredibly difficult, and players who manage to do so have focused on developing their offensive skills to the detriment of their defensive skills. This, in turn, makes them less capable defensively, which leads to more goals against. From this data it is impossible to say for certain what drives the mediating effect of *offense*, but it is an interesting and important avenue for future research.

With respect to the measurement model, both models sufficiently captured all the latent variables, as well as the structural model. However, only model 1 managed to fit the observed data as a whole. Going back to the CFA, we see that the largest standardized weight for the additional terms in model 2 is 0.64 for $A_{i}^{60}$, which is notably below the lowest standardized weight of 0.77 for *C*_*p*_ in model 1 (see Tables 5 & 7). Taken as a whole, these findings suggest that although a larger number of measured variables pertain to each latent variable, only a small number of variables that span raw, *τ*, and *δ* metrics are needed to sufficiently capture core concepts such as offense, defense, and possession, and that the majority of measured variables, fall under the purview of the disturbance terms.

In having identified a model that conveys the multivariate nature of hockey, and that is applicable across multiple seasons, we are able to not only generate factor scores for latent variables, but also combine these scores into an *overall* score. These scores, be they for *possession*, *offense*, *defense*, or *overall*, can then be used to rank players in a more nuanced way that if we were to rely on measured variables alone. Moreover, the ability to generate different *overall* scores by applying different weightings to latent variables allows us to prioritize components of player performance. Thus, if we wanted to identify the best overall player who also exhibits a high level of defensive responsibility, we could simply adjust latent variable weights to reflect this (e.g., *Overall* = *Possession*/10 + *Offense* + 2(*Defense*)).

## Supporting information

S1 File NHL data. Should interested parties want the corresponding R code, the author is happy to provided in upon request.
(XLSX)
